# An efficient coral survey method based on a large-scale 3-D structure model obtained by Speedy Sea Scanner and U-Net segmentation

**DOI:** 10.1038/s41598-020-69400-5

**Published:** 2020-07-31

**Authors:** Katsunori Mizuno, Kei Terayama, Seiichiro Hagino, Shigeru Tabeta, Shingo Sakamoto, Toshihiro Ogawa, Kenichi Sugimoto, Hironobu Fukami

**Affiliations:** 1 Graduate School of Frontier Sciences, The University of Tokyo, Kashiwanoha, Kashiwa, Chiba 277-8561 Japan; 2 Graduate School of Medical Life Science, Yokohama City University, 1-7-29, Suehiro-cho, Tsurumi-ku, Yokohama 230-0045 Japan; 3 RIKEN Center for Advanced Intelligence Project (AIP), 1-4-1 Nihonbashi, Chuo-ku, Tokyo, 103-0027 Japan; 4 RIKEN Medical Sciences Innovation Hub Program, 1-7-22 Suehiro-cho, Tsurumi-ku, Yokohama, 230-0045 Japan; 5 Windy Network Corporation, 896-1 Aoichi, Minamiizu, Kamo-Gun, Shizuoka 415-0151 Japan; 6 Faculty of Agriculture, University of Miyazaki, Miyazaki, Miyazaki, 889-2192 Japan

**Keywords:** Environmental sciences, Engineering

## Abstract

Over the last 3 decades, a large portion of coral cover has been lost around the globe. This significant decline necessitates a rapid assessment of coral reef health to enable more effective management. In this paper, we propose an efficient method for coral cover estimation and demonstrate its viability. A large-scale 3-D structure model, with resolutions in the x, y and z planes of 0.01 m, was successfully generated by means of a towed optical camera array system (Speedy Sea Scanner). The survey efficiency attained was 12,146 m^2^/h. In addition, we propose a segmentation method utilizing U-Net architecture and estimate coral coverage using a large-scale 2-D image. The U-Net-based segmentation method has shown higher accuracy than pixelwise CNN modeling. Moreover, the computational cost of a U-Net-based method is much lower than that of a pixelwise CNN-based one. We believe that an array of these survey tools can contribute to the rapid assessment of coral reefs.

## Introduction

Coral reefs play an important role in coastal environments throughout the world, providing food, resources and income to over 500 million people^1^, while supporting up to nine million species and a quarter of all marine life on Earth^2^. They also contribute to clean water, removing nitrogen and carbon, and constitute a natural barrier for coastal protection against hurricanes and storms. However, over the last 3 decades, up to 80% of coral cover has been lost in the Caribbean^1^ and up to 50% in the Indo-Pacific^3,4^, largely due to anthropogenic stressors that include over-fishing, pollution, sedimentation, habitat destruction and climate change^5–7^. An intensive analysis of the extent of coral reef loss and decline in growth was conducted by Pratchett et al.^8^. This grave decline requires techniques to rapidly assess coral reef health to enable more effective management and the development of effective conservation strategies^9^.

Various methods have been developed for monitoring benthic marine habitats such as coral reefs. In general, field transects, such as line intercept transects (LITs), photo line intercept transects (PLITs) and video transects (VTs) have been the most widely used methods, as they are simple to conduct and relatively inexpensive^10–13^. However, these *in-situ* visual methods entail long sampling times due to their small-scale scope, are limited by factors such as diver air tank supply and pose varying degrees of associated risk. To overcome these problems, marine biologists and ecologists have increasingly come to rely on imagery obtained from platforms such as autonomous underwater vehicles (AUVs) or remotely-operated vehicles (ROVs) for marine monitoring^14–17^. Such platforms can collect a large number of images, while the total data handling size concurrently increases with technological progress. As a result, much time and effort must be devoted to obtaining ecological data from the collected images, such as the extent of coral reefs and seagrass meadows^18^. With recent advancements in computer imaging technologies and growing interest in the topic within the scientific community, a huge amount of data on coral reefs is being collected and the manual analysis of images by humans is no longer practical^17,19,20^. In recent years, convolutional neural networks (CNNs) have shown outstanding accuracy in automatic image classification and segmentation^21,22^, especially in the field of computer vision. Several studies in the literature have applied variants of the CNN method to coral classification or segmentation using various types of dataset, e.g., those obtained by laboratory experiments or by divers and underwater vehicles^23–25^. However, research using large-scale images obtained from the sea remains limited^25^ and continuous research effort to remedy this is required.

**Figure 1:**
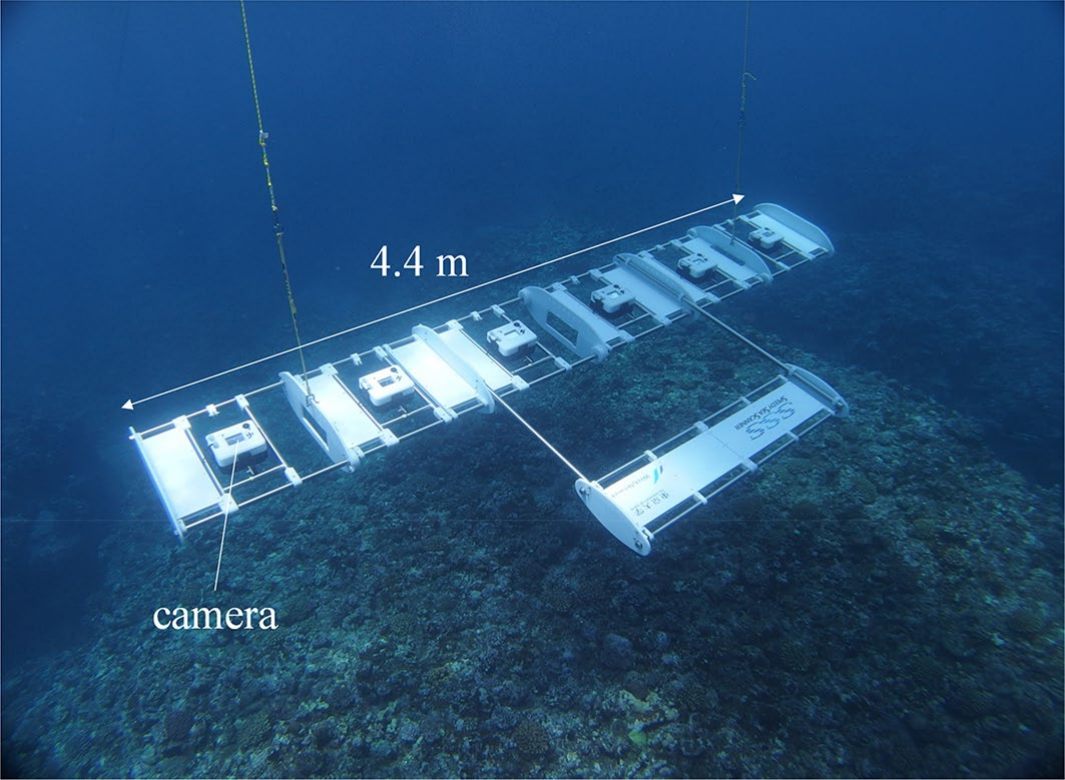
Speedy Sea Scanner (SSS). Six cameras reside on the towed body. The attitude is maintained by the tailplane.

Through recent technology innovation, a more efficient image collection system, namely the “Speedy Sea Scanner (SSS)” (Fig. 1), which is a towed optical camera array that has succeeded in making a large-scale and high-resolution 2-D image (orthophoto) of the seafloor around the Kujuku islands in 2017^26^. When that imaging was collected, the surveying efficiency of the SSS was approximately 7,000 m^2^/h. According to previous studies, the surveying efficiency of divers or swimmers is approximately 150 m^2^/h^12,13^ while that by AUVs is some 2,470 m^2^/h at 2 m above the seafloor^27^. Thus, the surveying efficiency of the SSS is a dramatic improvement over these earlier methods and we can now obtain a large number of images with greater ease than before. In addition, precise depth information on the seafloor can be obtained from a 3-D structure model derived from part of the survey area^26^. However, a large-scale 3-D structure model of an entire survey area has not been generated and the accuracy of the seafloor’s depth distribution has yet to be evaluated. In conjunction with the SSS technology’s development, to reduce the time required for the analysis of huge quantities of data, an automatic coral coverage estimation method that makes use of conventional image segmentation approaches based on pixelwise CNN and bag-of-visual-words (BoVW) was proposed and the performances were compared^26^. In the comparison, the accuracy of pixelwise CNN was found to be better than that of BoVW. However, field sampling data is still lacking and a problem in the form of the substantial computation cost of large-scale coral cover estimations was encountered, undermining their practical application.

In this study, we demonstrate the effectiveness of the coral cover estimation method we propose herein. We collected seafloor images using the SSS off the coast of Kumejima in Okinawa, Japan, and used them to construct a large-scale 3-D model (See the Methods section for the methodological details of SSS). In addition, we obtained multibeam echosounder (MBES) data to use as reference data for the seabed topography. In general, the MBES data, where collected and available, feeds into the General Bathymetric Chart of the Oceans (GEBCO) to generate wider-scale bathymetric data sets for the entire ocean (https://www.gebco.net/). Therefore, we prepared the two digital elevation models (DEMs) attained from the SSS and MBES data. Herein, we refer to them as DEM_SSS_ and DEM_MBES_, respectively. The resolution on the horizontal plane and the accuracy of depth information in the vertical plane within the DEMs was then compared. We show that the resolution in DEM_SSS_ is much higher than that in DEM_MBES_ and quantify the difference between the two.

In addition, we propose another segmentation method based on U-Net^28^, (which is often used in medical applications)^29^, and perform the coral cover estimation using the large-scale 2-D image (orthophoto) converted from the 3-D structure model. The computational cost of the U-Net-based segmentation method is much smaller than that of the pixelwise CNN-based one^26^. The prediction time of U-Net is about 1/1,000 for pixelwise CNN (See the Results section for the details). We believe that an array of these survey tools can contribute to enabling the rapid assessment of coral reefs.

## Methods

### Data collection

The SSS towed optical camera array system was used for collecting the images. The following is a brief list of the general advantages of the SSS:

- Lower cost of development and maintenance than that for underwater vehicles.
- Higher surveying efficiency than that which can be achieved by methods relying on divers and underwater vehicles.
- Simple operation without additional electrical equipment.
- Robust pair-matching between adjacent images for 3-D structure model generation.
- High portability—it can be carried by a small boat and easily deployed at the survey site, including small islands.

The system’s depth rating is 50 m. The length of the array’s baseline is 4.4 m, with six equally spaced cameras (Panasonic DC-GH5 with custom-made waterproof housing and batteries) installed on the platform. Each optical camera can record up to 6 h of high-definition video at a recording rate of 23.98 frames per second. We determined the length such that two adults could handle the system and carry it to the survey area by small boat. The attitude during towing is held stable by the tailplane and the tilt angle can be tuned through the attachment position of the towing rope. The system was towed by the survey boat, which was equipped with a navigation system (POS MV, Applanix). The positioning error of the navigation system was approximately ± 1 m. The distance of the SSS from the seafloor was set to around 2–5 m, while the boat maintained a speed of 2–3 knots during the survey. To keep the safety of survey, and monitor the vertical position of SSS, a fish echosounder (HDS Gen2, LOWRANCE) was equipped on the ship. In addition to the SSS survey, precise seabed topography was measured using multi-beam sonar (Sonic 2022, R2Sonic LLC) with an operating frequency of 400 kHz. We also used bathymetric data to validate the accuracy of the depth distribution in the 3-D structure model (DEM_SSS_) generated from the collected images. The DEM_MBES_ was generated from the sounding data using the commercial software (HYPACK, Xylem Inc.). The tidal and sound refraction corrections were conducted following the general processing flow in the software. The sound profile for the sound refraction correction was measured by the Conductivity Temperature Depth profiler (CTD; Minos.X, AML Oceanographic Ltd.) before the survey. The vertical resolution of the multi-beam sonar was 1.25 cm with 0.9° × 0.9° directivity. The mean density of the sound data in a grid was 7.77 and we adopted the central value to the DEM_MBES_ grid data.

The images were collected offshore at Kumejima, Okinawa prefecture, Japan on July 6, 2018. Kumejima is surrounded by a wide variety of different marine habitats, e.g., intertidal mudflats and rocky shores, vibrant coral reefs, muddy/sandy substrates and submarine limestone caves. The SSS survey was conducted in an area with water depths spanning 5–45 m. The offshore survey time taken at Kumejima was about 56 min for the seven survey lines.

### Large-scale 3-D structure model generation

Details of the data processing methods employed were outlined in our previous study^26^. Here, we recall in brief the image processing flow. First, the GPS device and cameras were time-synchronized with GPS time. Next, continuous still images were obtained from the video data. In this study, we extracted two still images per second. Color corrections were then performed on the images. The camera locations were estimated on the basis of GPS data and added to the corresponding still images. The GPS data was then up-sampled using the cubic spline interpolation method. In this case, the up-sampling rate was 10 times that of the original data points. Here, the vertical distance between the fish echosounder and the SSS was recorded using a fish echosounder with 0.1 m vertical resolution; then, the tidal correction was conducted to the vertical distance. Also, the vertical offset between the water surface and the fish echosounder was directly measured by measure. In addition, we directly measured the horizontal distance between the GPS and the SSS, during the survey. With using the measured distances, the position offset of the SSS was corrected. A 3-D point cloud was reconstructed from the continuous images using a low-cost commercial software (Metashape, Agisoft) employing Structure from Motion (SfM) techniques. SfM is a technique that utilizes 2-D image series to construct a 3-D structure model^30,31^. From the 3-D structure model, the DEM_SSS_ and 2-D image (orthophoto) can be produced.

### Network architecture

We built a U-Net-based^28^ deep neural network that takes an image of 512 × 512 pixels as input and produces a predicted label image of the same size (see the supplementary Fig. S1). This network, like the U-Net, consists of an encoder part in the first half and a decoder part in the second. The encoder network extracts a small feature map from the input image using the convolution (Conv) and pooling (Pool) layers, while the decoder expands to the original image size using the convolution and up-sampling (Upsamp) layers. The encoder block consists of two repeating layers consisting of 3 × 3 convolutions and a 2 × 2 maximum pooling with two strides for the rectified linear unit’s (ReLU) activation. The decoder block comprises 2 × 2 up-sampling and two 3 × 3 convolution layers. After each of the first three decoder blocks, a 50% dropout layer was added. In the final layer of the decoder, the feature map was converted into the two classes (coral or non-coral) by a 1 × 1 convolution and then a softmax activation function was applied. The skip connection bridges the gap between each convolution layer of the encoder and a corresponding up-sampling layer of the decoder in order to preserve high-resolution information from the input image. The skip connection simply concatenates the channels in each layer of the encoder with one from the decoder. We implemented the above network using the Keras^32^ library with the Tensorflow^33^ backend.

### Network training and evaluation

For the training of the network, a data-augmentation technique based on rotation^21,34^ was employed to improve prediction performance and, in particular, to prevent overfitting. In the images used in this study, there is no specific orientation and the coral remains even when rotated. Thus, the rotated images at 90, 180 and 270 degrees and the corresponding labeled coral images were used in the training.

When training the U-Net and pixelwise CNN models, we used the F-measure score as a loss function and maximized the loss in order to train the networks. We employed Adam^35^, a variant of the mini-batch Stochastic Gradient Descent (SGD) solver^36^ for training the network and explored the optimal hyperparameters within the following ranges: learning rates of SGD of 10^−4^, 10^−3^ and 10^−2^ and epoch numbers of 100, 200 and 300, respectively. We fixed the batch size to 4.

For the evaluation of the prediction performance, we performed a five-fold cross-validation^37^. That is, the 200 images of the dataset were randomly divided into five sub-datasets and then four of these were used to train the U-Net. The sub-datasets that were not used for training were evaluated by accuracy, precision, recall and the F-measure. The five cross-validation scores were calculated by averaging the five training and evaluation sessions with different training sub-datasets.

### Evaluation metrics

We employed four evaluation metrics, namely accuracy, precision, recall and the F-measure, to evaluate the prediction performances of the U-Net and pixelwise CNN models. The accuracy was defined as the ratio of successfully predicted pixels to all of the predicted pixels. Although this metric indicates overall performance, it is not a suitable measure when the percentage of coral is very low or high. For example, when the percentage is very low, the model that predicts all pixels as non-coral showed high accuracy. Therefore, we also calculated the F-measure for evaluation using precision and recall. Precision is the fraction of manually-labeled pixels such as coral amongst the pixels predicted to be coral. Recall is the fraction of relevant pixels that were successfully predicted to be coral. Finally, the F-measure is defined as the harmonic mean of precision and recall as follows:

$${\text{F-measure}} = \frac{{2 \cdot {\text{Precision}} \cdot {\text{Recall}}}}{{{\text{Precision}} + {\text{Recall}}}}.$$

when the values of precision and recall are high on balance, the F-measure also reaches a high value. The ranges of the four metrics are 0 to 1.

## Results and discussion

### Reconstructed optical map of the seafloor

**Figure 2:**
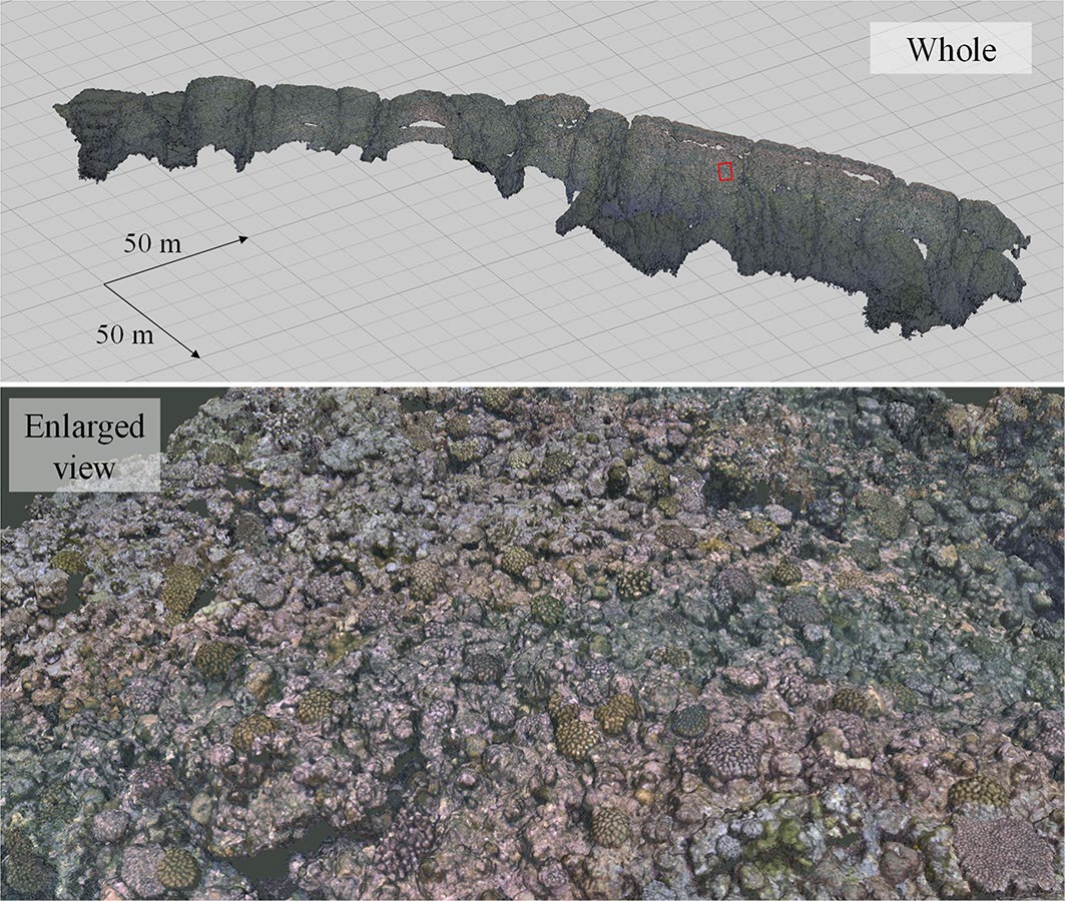
3-D structure model: the top is a whole view and the bottom an enlarged view of the inside of the red rectangle (above).

The 3-D structure model was generated from 30,957 images obtained across seven survey lines (Fig. 2). The total length of each survey line was around 1,838 m. The resolutions of the x, y and z axes were 0.01 m and the corals can be identified from the constructed model. The survey site is a well-known diving spot and we can identify some drop-offs with depth differences of around 5–7 m.

**Figure 3:**
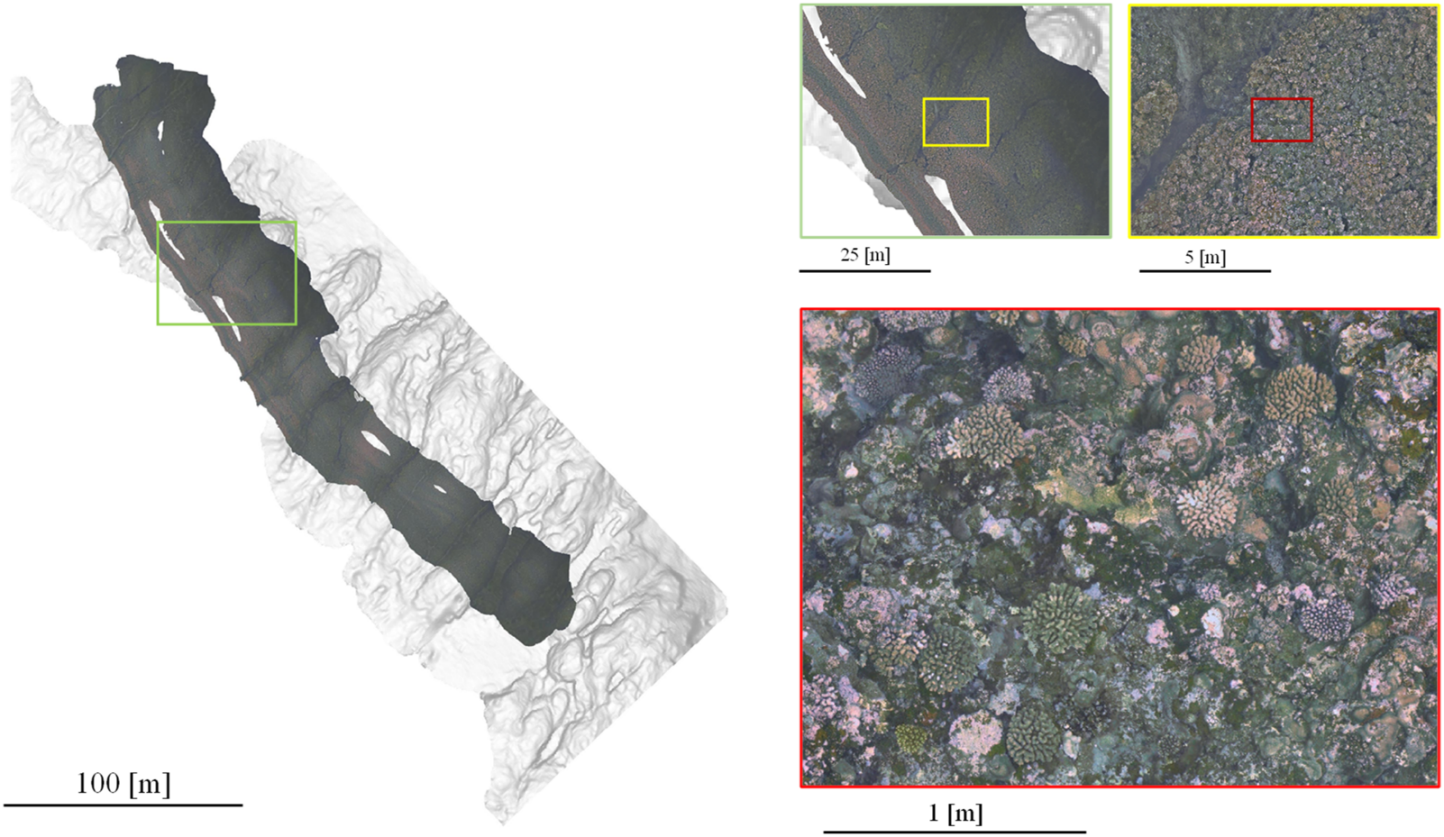
The 2-D image (orthophoto) at various scales. The 2-D image is overlaid on the hill-shaded topography generated from MBES data. The survey was conducted in the northern coastal area of Kumejima.

The large-scale 2-D image was produced from the 3-D structure model and is illustrated in Fig. 3. A survey area of 11,434 m^2^ was covered, yielding a calculated survey efficiency of 12,146 m^2^/h. The pixel resolution in the horizontal plane (x–y plane) is about 3.5 mm/pixel (± 0.4%); the viewing scale can be adjusted on any type of commercial or free geographical information system (GIS) software. As shown in the Fig. 3, the resolution was enough to identify the coral. We can identify a large quantity of coral from the high-resolution image in Fig. 3 and the presence of at least 10 individual species of corals, such as *Pocillopora eydouxi* and *P. verrucosa*, are confirmed in this data by the expert.

**Figure 4:**
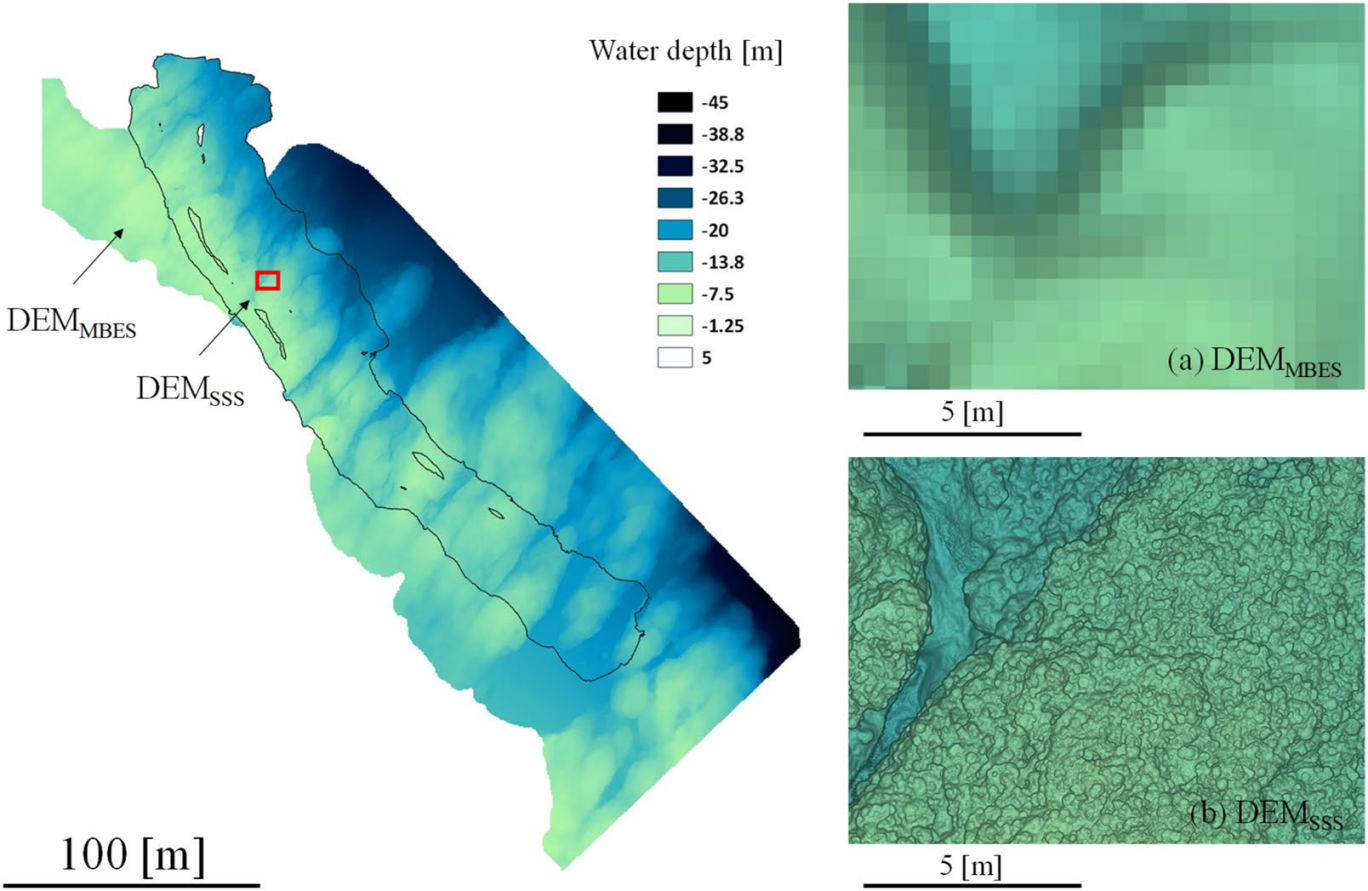
Combined DEM. DEM_SSS_ (inside the black border) is overlapped onto the DEM_MBES_. The right-hand images comprise an enlarged view of the same location (red rectangle).

**Figure 5:**
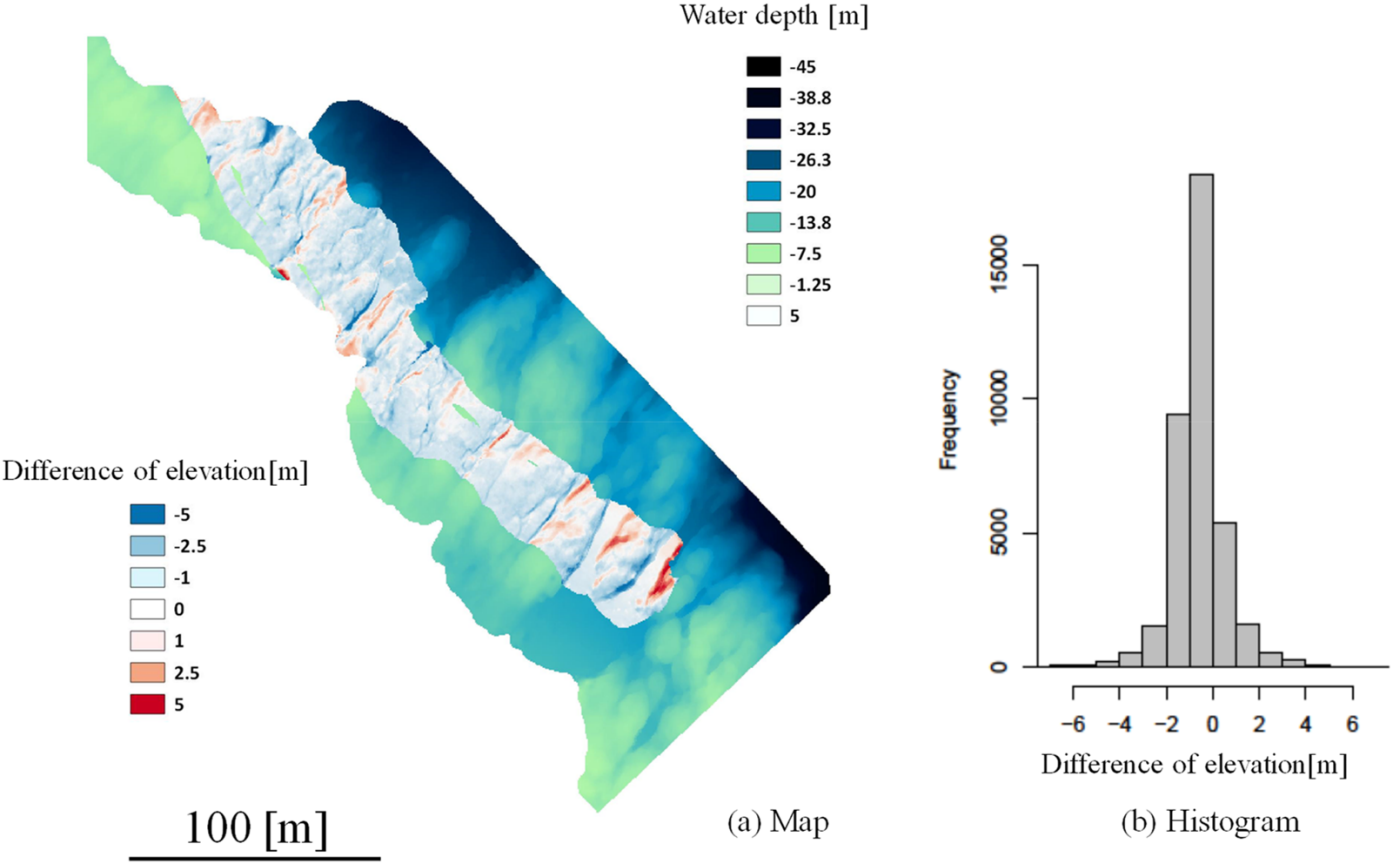
Left: distribution map of the elevation difference. Right: histogram of the elevation difference at the pixel level.

In addition, the DEM_SSS_ inside the black border line was produced from the 3-D structure model and overlapped onto the DEM_MBES_ (background), as is shown in Fig. 4. It seems that the connection between the DEM_SSS_ and DEM_MBES_ is seamless. To compare the DEM resolutions, enlarged figures are illustrated in Fig. 4a,b. The resolution of the image (horizontal plane) in Fig. 4a is 0.5 m/pixel and in Fig. 4b is 0.01 m/pixel; thus, we can extrapolate the seafloor structure with precision using the DEM_SSS_. The accuracy by the photogrammetry method was well discussed in the literature (approximately 1–2 mm at 3 m distance)^38^. The distribution of differences of depths in the vertical plane (elevation) was calculated and is illustrated as the color gradation in Fig. 5a. From this figure, it can be seen that the difference around the slope area is large. In addition, Fig. 5b shows a histogram of this difference [− 0.68 ± 1.16 m (mean ± S.D., n = 38,602)] and slightly shifts to the left (minus direction). This means that the DEM_SSS_ tends to become lower than the DEM_MBES_. The supplementary Fig. 2 shows the locations of the Ground control points (GCP) in DEM_MBES_ and DEM_SSS_ to validate the difference of depths [1.61 ± 0.14 m in the horizontal plane, 0.74 ± 0.11 m in the vertical direction (mean ± S.E., n = 21)]. The GCPs were arbitrarily picked up from the point data at the characteristic land features. From these results, the error was larger in the horizontal plane than in the vertical direction. We assume the main difference of depths was caused by the gap in the horizontal plane due to the GPS positioning error (± 1 m).

Although a slight difference in the vertical plane is observed, this high-resolution DEM_SSS_ will offer useful information for the advanced surveying of seabed topography, especially in shallow coastal areas. This precise seabed topography will contribute not only to coral surveys but also to other ecological, engineering and geographical studies, e.g., high-resolution advection modeling and structural calculations of natural reefs^39–41^. The survey efficiency of 12,146 m^2^/h achieved in this study is higher than the 7,000 m^2^/h of the previous study^26^, because six cameras were used in this case compared to five in the previous one due to battery problems. In addition, the water transparency was better than before (see the supplementary Fig. S3); therefore, we could maintain the SSS at a high altitude of around 3–5 m. Thus, the efficiency of the SSS is at least five times greater than that of an AUV and some 80 times higher than that of diving, making it suitable for the rapid assessment of coral reefs.

Of course, the condition is different in each survey site; therefore, we should search the optimal survey strategy to fit them. The use of the acoustic positioning system or the already-known benchmark position on the sea floor will become one of the solutions to keep the accuracy of the DEM_SSS_. Also, in case of the deeper sea survey or more turbid condition, we should use the LED lights and care the safety of the operation of the towed camera array system with long towing rope to avoid hitting the corals.

### Evaluation of U-Net-based segmentation

**Figure 6:**
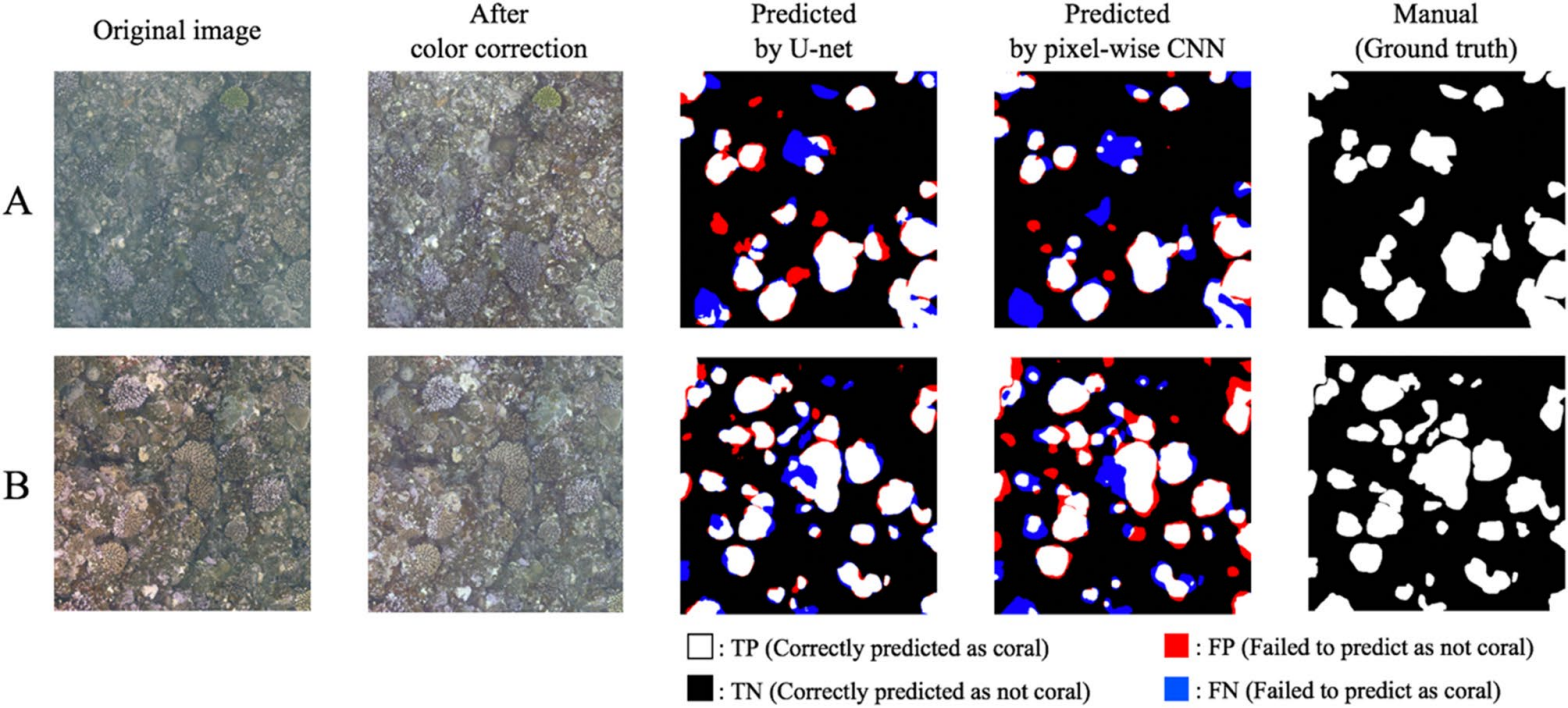
Prediction examples by U-Net and pixelwise CNN. The images in the leftmost column are original images; the second column comprises images processed by color labeling; the third and fourth columns are prediction results by U-Net with CC and DA and pixelwise CNN (window = 64 × 64 pixels), respectively; the white areas in the rightmost column show the manually-labeled coral areas.

In this study, we propose and evaluate a U-Net-based coral segmentation approach for the efficient surveying of large areas, such as depicted in Fig. 3. (See the Methods sections for details of the U-Net model and data processing). For training and evaluation, we divide the entire dataset (Fig. 3a) into 14,016 images of 512 × 512 pixels. Each divided image measures about 3.2 m^2^. We randomly selected 200 images from those divided and manually labeled images of coral under the supervision of coral experts. The images in the leftmost and rightmost columns in Fig. 6 are examples of the divided images and labeled coral images, respectively.

We then performed training and performance evaluations of the dataset of the 200 image pairs above. The processing of the color correction (CC)^26^ and data-augmentation (DA) for the obtained images, which was based on rotations^21,34^, may affect prediction performance. Therefore, we trained and evaluated four types of U-Net models with and without CC and DA, respectively. Furthermore, to compare prediction performance with the U-Net model, we employed the pixelwise CNN model, which had exhibited good performance in our previous work^26^. We evaluated the performances of the pixelwise CNN models with different input window sizes of 32 × 32, 48 × 48, 64 × 64, 96 × 96, 128 × 128 and 160 × 160, because the size of the local images used for the input window of the pixelwise CNN model greatly influences the prediction performance. (See the Methods section for details of the training procedure and evaluation metrics).

Figure 6 shows prediction examples for two test images, A and B. The images in the leftmost column are the original ones, while the images in the second column were processed by color correcting the originals. The images in the third and fourth columns are the predicted results using U-Net with CC and DA and the pixelwise CNN (window: 64 × 64 pixels) models, respectively. The results for the different processing conditions (CC and DA) of the U-Net model are shown in Fig. S1. The black and white areas indicate pixels that were successfully predicted as coral (TP: True Positive) and non-coral (TN: True Negative) areas, respectively. On the other hand, the red and blue areas were those that were wrongly predicted as coral (FP: False Positive) and non-coral (FN: False Negative), respectively. The white area in the rightmost column shows the manually-labeled coral area. The prediction accuracies for images A and B were, respectively, 0.913 and 0.924 with U-Net and 0.903 and 0.870 with pixelwise CNN. Both methods achieved a high degree of accuracy of about 0.9, but U-Net showed slightly better performance. In addition, the F-measures for images A and B were 0.805 and 0.857 with U-Net and 0.759 and 0.763 with pixelwise CNN. These results suggest that U-Net has the potential to identify corals with greater accuracy than pixelwise CNN.

**Figure 7:**
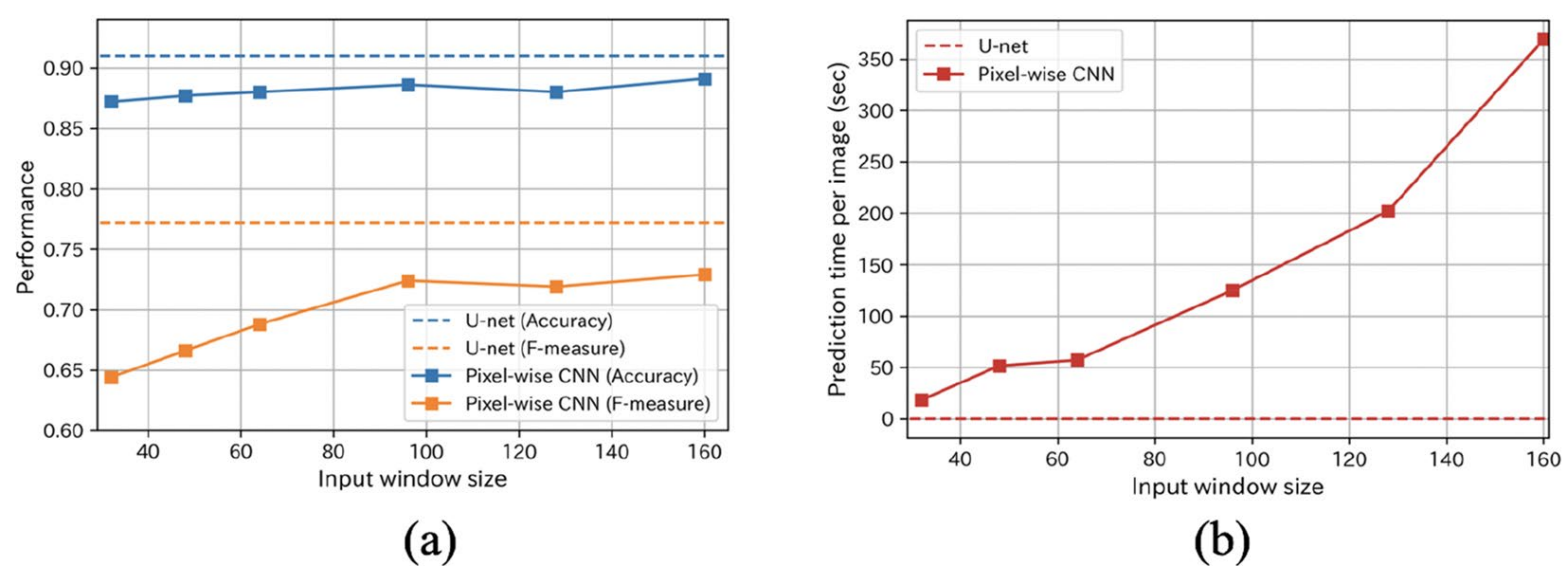
The relationship between prediction performance and prediction time: (**a**) the dotted lines correspond to the accuracy (blue) and F-measure (orange) of U-Net with CC and DA. The blue and orange lines show the accuracy and F-measure of pixelwise CNN with different window sizes; (**b**) Prediction time per image (512 × 512 pixels) using U-Net and pixelwise CNNs. The dashed line indicates the prediction time by U-Net, which was 0.057 s.

To evaluate the performances of U-Net and pixelwise CNN in more detail, we conducted evaluations using a dataset of 200 labeled images based on a five-fold cross-validation. (See the Methods section for methodological detail on this validation). Table 1 and Fig. 7a show the evaluated performances of U-Net with and without CC and DA, as well as pixelwise CNN using the images with CC and DA with different window sizes. The predictions by all variants of U-Net achieved high levels of accuracy (> 0.9). From the results listed in Table 1, it can be confirmed that performance tends to increase with the application of CC and DA. The U-Net model with both CC and DA showed the highest accuracy (0.910) and F-measure (0.772). The pixelwise CNN result shows that the performance tends to increase with increasing window size. However, it is clearly shown in Fig. 7a that the accuracy (blue-dashed line) and F-measure (orange-dashed line) of the U-Net exhibit better performances compared to that of the pixelwise CNN. These results indicate that the U-Net has high predictive performance, and both CC and DA are effective for improving this. While pixelwise CNN uses the local information of window sizes as its main input for prediction, U-Net utilizes the global information of the entire input image (see supplementary Fig. S1). Therefore, U-Net is considered to have achieved higher performance than pixelwise CNN.

**Table 1:** Performances of U-Net and pixelwise CNN based on five-fold cross-validation.

	Accuracy	Recall	Precision	F-measure
U-Net w/o CC and w/o DA	0.901	0.710	0.785	0.740
U-Net w/o CC and w DA	0.908	0.748	0.791	0.763
U-Net w CC and w/o DA	0.902	0.718	0.783	0.743
U-Net w CC and w DA	0.910	0.767	0.788	0.772
Pixelwise CNN (input size: 32 × 32)	0.872	0.586	0.742	0.644
Pixelwise CNN (input size: 48 × 48)	0.877	0.614	0.745	0.666
Pixelwise CNN (input size: 64 × 64)	0.880	0.656	0.750	0.688
Pixelwise CNN (input size: 98 × 98)	0.886	0.752	0.711	0.724
Pixelwise CNN (input size: 128 × 128)	0.880	0.733	0.714	0.719
Pixelwise CNN (input size: 160 × 160)	0.891	0.739	0.739	0.729

We assessed the details of the relationship between prediction performance and prediction time. Figure 7b displays prediction times per image using U-Net and pixelwise CNN with different window sizes. We used an Nvidia GeForce GTX 1,080 Ti GPU with an Intel Xeon CPU E5-2,630 v4 computing core. These results indicate that the prediction time rapidly increases as the input size expands, while the prediction time of U-Net is very short (0.057 s). Note that the prediction time of U-Net does not change because the input size is constant (512 × 512 pixels). The prediction time of U-Net is about 1/1,000 for pixelwise CNN with a window size of 64 × 64. The results shown Fig. 7a,b indicate that U-Net-based prediction is more accurate and substantially faster than pixelwise CNN.

### Estimation of coral cover in the surveyed area

**Figure 8:**
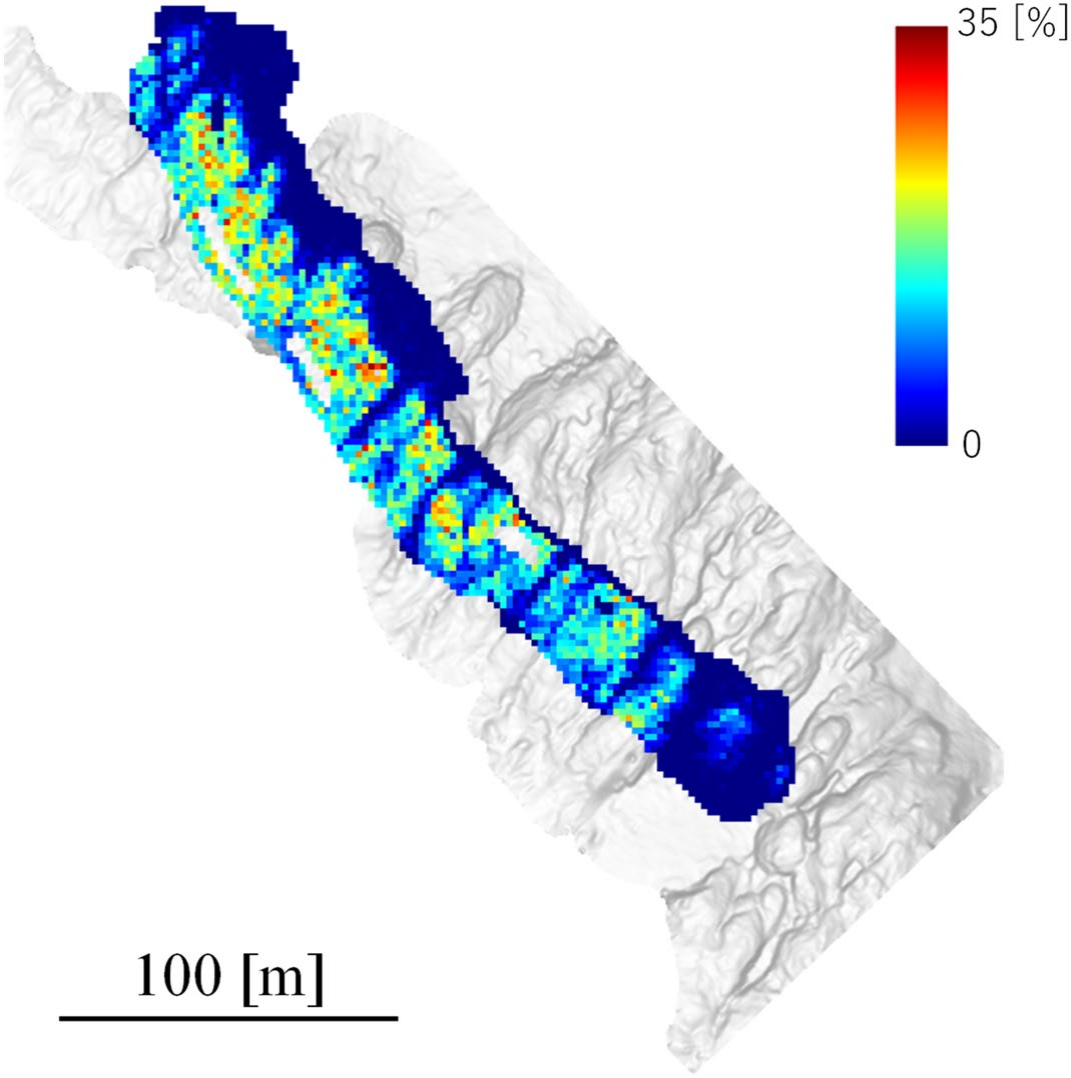
Distribution map of coral cover prediction. The color gradation shows the percent coral cover.

We built a prediction model for the entire surveyed area using all 200 images and the U-net with CC and DA, which had exhibited the best performance in the above evaluations. The 2-D image (orthophoto) of the entire surveyed area was divided into 14,016 local images (512 × 512 pixels). We estimated the quantity of coral in the surveyed area (11,434 m^2^) using the built model and divided the images. The calculation time for this estimation was 1,120 s (18.7 min) using the same GPU and CPU as that outlined above. Figure 8 shows the overall coral coverage prediction by the model. The predicted percent coral cover was distributed from 0 to 35%. According to the previous survey, conducted in 2011 by scuba divers using the manta-method, the coral cover in the area was estimated to be around 25 to 50%^42^. The results this time around were about half of what they were last time, so our results indicate a decline in coral cover, which may be due to the 2016 bleaching event^43^. As previously described, the changes to coral reefs have been dramatic and determining the mechanisms underlying these requires the capacity to rapidly assess reefs. In addition, the U-net based segmentation method has the possibility to be applied for the species cover, or disease prevalence studies. Although the fields are different, Saito et al. have classified the layers of two-dimensional materials into three classes^44^. Also, Kohl et al. have classified images of street scenes taken from a camera into 19 classes, including person, car, and road^45^. As remarked above, the efficient survey method presently under discussion has the potential to become a useful tool for quantitatively investigating biological systems such as coral.

## Conclusions

In this paper, we proposed an efficient method for coral cover estimation and demonstrated its viability. A large-scale 3-D structure model, with resolutions in the x, y and z planes of 0.01 m, was successfully generated by means of a towed optical camera array system (Speedy Sea Scanner). The survey efficiency attained was 12,146 m^2^/h. In addition, we propose a segmentation method utilizing U-Net architecture and estimate coral coverage using a large-scale 2-D image. The U-Net-based segmentation method has shown higher accuracy than pixelwise CNN modeling. Moreover, the computational cost of a U-Net-based method is much lower than that of a pixelwise CNN-based one. We believe that an array of these survey tools can contribute to the rapid assessment of coral reefs.

## Supplementary information

Supplementary Information 1.
