# Supplementary material for: An efficient coral survey method based on a large-scale 3-D structure model obtained by Speedy Sea Scanner and U-Net segmentation
Source: Sci Rep. 2020 Jul 31;10:12416. doi: 10.1038/s41598-020-69400-5 (PMC7395762; doi:10.1038/s41598-020-69400-5)
Supplement: Supplementary file 1 — Supplementary Information 1. [file 41598_2020_69400_MOESM1_ESM.pdf]

Supplementary Information for
An efficient coral survey method based on a large-scale 3-D structure model obtained by Speedy Sea Scanner and U-Net segmentation

Katsunori Mizuno[1,*], Kei Terayama[2,a,b], Seiichiro Hagino[1], Shigeru Tabeta[1], Shingo Sakamoto[3], Toshihiro Ogawa[3], Kenichi Sugimoto[3], Hironobu Fukami[4]

[1]Department of Environment Systems, Graduate School of Frontier Sciences, The University of Tokyo, Kashiwanoha, Kashiwa, Chiba 277-8561, Japan

[2]Graduate School of Medical Life Science, Yokohama City University, 1-7-29, Suehiro-cho, Tsurumi-ku, Yokohama 230-0045, Japan

[a]RIKEN Center for Advanced Intelligence Project (AIP), 1-4-1 Nihonbashi, Chuo-ku, Tokyo 103-0027, Japan

[b]RIKEN Medical Sciences Innovation Hub Program, 1-7-22 Suehiro-cho, Tsurumi-ku, Yokohama 230-0045, Japan

[3]Windy Network Corporation,

6-4-25 Magarikane, Suruga-ku, Shizuoka, Shizuoka 422-8006, Japan

[4]Faculty of Agriculture, University of Miyazaki,

Miyazaki, 889-2192, Japan

***Corresponding author: Katsunori Mizuno**

kmizuno@edu.k.u-tokyo.ac.jp

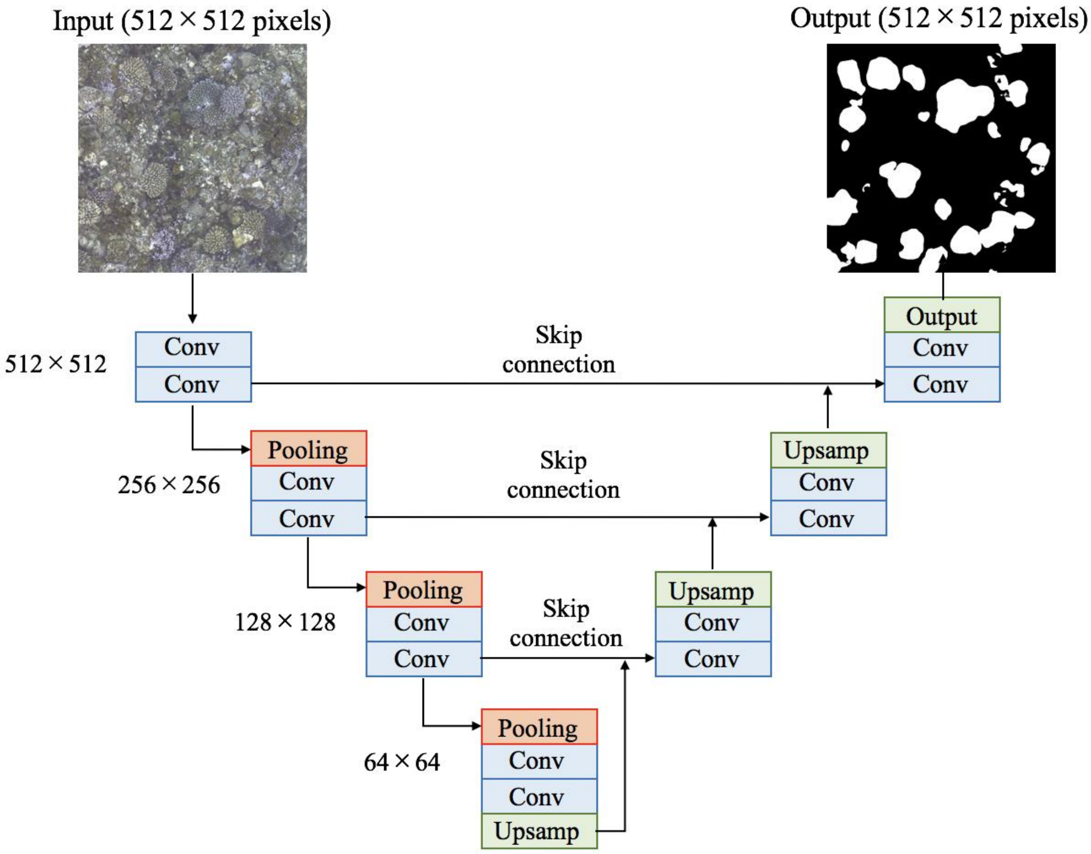

Supplementary Fig. S1. Overview of the U-Net-based coral prediction network.

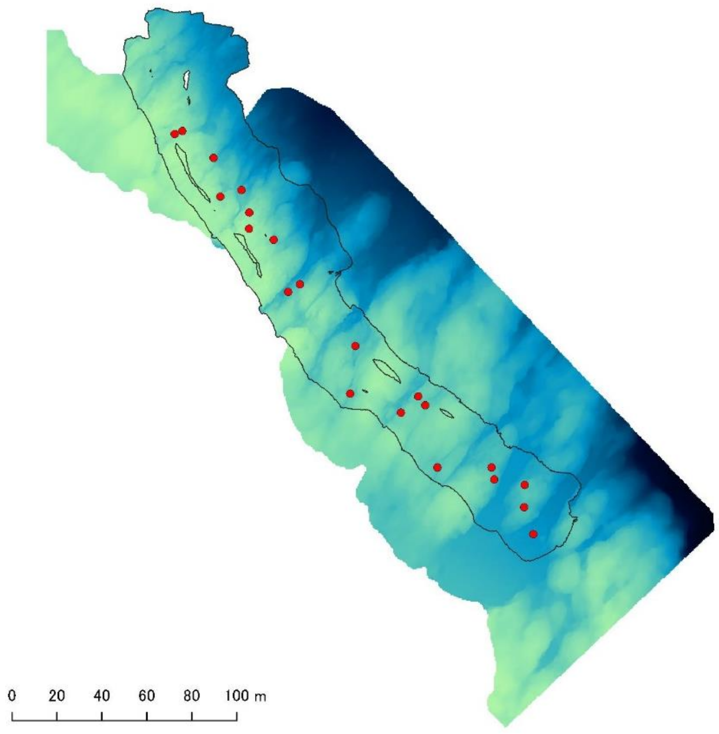

Supplementary Fig. S2. The locations of GCPs on the DEM.

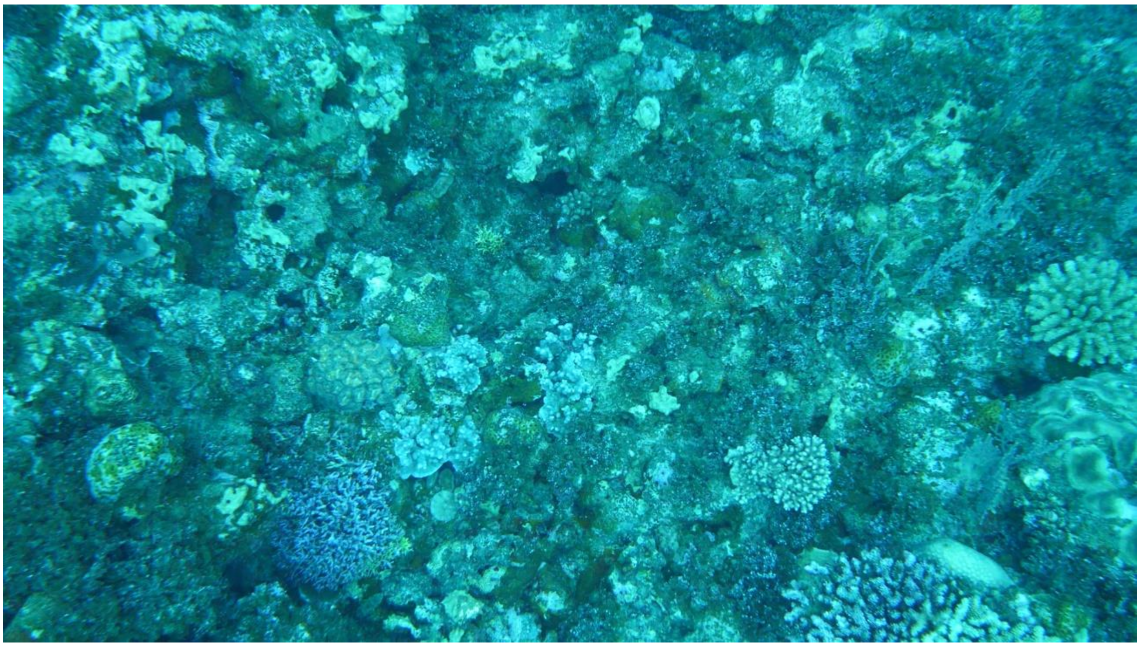

(a) RAW image captured in the present survey (at the coast of Kumejima) with 3 m altitude at 20 water depth.

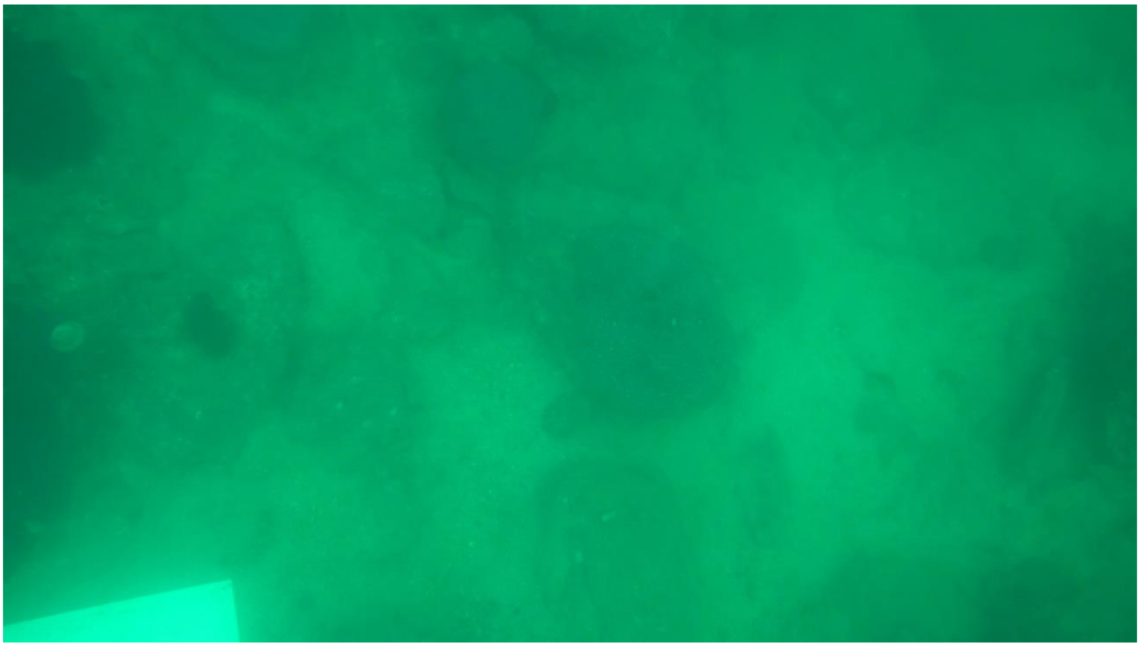

(b) RAW image captured in the past survey (at the coast of Kujuku islands) with 3 m altitude at 20 water depth

Supplementary Fig. S3. Raw images captured in (a) the present survey (at the coast of Kumejima) and (b) the past survey (at the coast of Kujuku islands).
